# Radiological and Clinical Evaluation of the Ipsilateral Ankle of Patients Who Underwent Total Knee Arthroplasty: A Prospective Study

**DOI:** 10.3390/jcm15176854

**Published:** 2026-09-04

**Authors:** Emre Tosun, Özal Özcan, Aylin Yücel, Serkan Savaş, Sakhi Ahmad Fazli

**Affiliations:** 1 Department of Orthopedics and Traumatology, Denizli Servergazi State Hospital, 20000 Denizli, Türkiye; 2 Department of Orthopedics and Traumatology, Faculty of Medicine, Afyonkarahisar Health Sciences University, 03030 Afyonkarahisar, Türkiye; 3 Department of Radiology, Faculty of Medicine, Afyonkarahisar Health Sciences University, 03030 Afyonkarahisar, Türkiye; 4 Department of Orthopedics and Traumatology, Çankırı Çerkeş State Hospital, 18600 Çankırı, Türkiye; 5 Department of Orthopedics and Traumatology, Faculty of Medicine, Lokman Hekim University, 06530 İstanbul, Türkiye

**Keywords:** total knee arthroplasty, ipsilateral ankle, mechanical alignment, talar tilt, bone marrow oedema, osteochondral lesion, magnetic resonance imaging, Foot and Ankle Outcome Score

## Abstract

**Background/Objectives:** Total knee arthroplasty (TKA) corrects coronal malalignment at the knee, but its effect on the structural status of the ipsilateral ankle is poorly characterised. This prospective study evaluated clinical, radiographic, and MRI-detected changes in the ipsilateral ankle after TKA, and tested whether such changes are related to the magnitude of mechanical axis correction. **Methods:** Seventy-one consecutive patients (80 operated limbs; mean age 63.9 ± 7.2 years; 83.1% female) undergoing primary TKA with a systematic neutral mechanical alignment strategy were assessed prospectively. Mechanical axis (MA) and talar tilt (TT) were measured on full-length weight-bearing radiographs, ankle-specific status was evaluated with the Foot and Ankle Outcome Score (FAOS), and ipsilateral ankle MRI was performed preoperatively, in the early postoperative period, and at follow-up (median 37 days, interquartile range (IQR) 34–44). Chondral/osteochondral findings and bone marrow oedema were recorded separately at the talus, distal tibia, and subtalar joint. Radiographic and MRI outcomes were analysed at the limb level using linear mixed-effects models and logistic generalised estimating equations with patient as the clustering variable; clinical outcomes were analysed at the patient level. **Results:** MA improved from 11.62 ± 5.57° to 4.29 ± 4.27° (mean change −7.34°, 95% confidence interval (CI) −8.49 to −6.18; *p* < 0.001), whereas TT was unchanged (−0.02 ± 1.87° to −0.22 ± 1.91°; *p* = 0.256). All FAOS subscales improved significantly (total 66.7 ± 27.6 to 84.4 ± 25.1; *p* < 0.001). Chondral/osteochondral findings were present in 41.2% of limbs preoperatively and were newly reported during follow-up in 40.4% of previously unaffected limbs. Newly reported bone marrow oedema was most frequent in the distal tibia (16/79 limbs, 20.3%), compared with the talus (8/79, 10.1%) and the subtalar joint (6/70, 8.6%). The magnitude of MA correction was not associated with change in TT (r = −0.040), change in FAOS (r = 0.023), or the development of new MRI findings (odds ratio (OR) 1.021 per degree; *p* = 0.682), and no dose–response gradient was observed across tertiles of correction. **Conclusions:** Following TKA with neutral mechanical alignment, substantial correction of the mechanical axis and significant clinical improvement occurred without a measurable change in talar tilt. MRI changes in the ipsilateral ankle were modest, concentrated in the distal tibia, and unrelated to the magnitude of alignment correction, suggesting that perioperative rather than alignment-related factors may predominate in the early postoperative period. Given the high preoperative burden of ankle degeneration and the short follow-up interval, the mechanisms and long-term significance of these findings remain uncertain.

## 1. Introduction

Knee osteoarthritis (OA) is the most prevalent form of large-joint degenerative disease and a leading global cause of chronic pain and disability. In 2021, an estimated 374.7 million people worldwide were living with knee OA, and the age-standardised prevalence has risen steadily over the past three decades [1]. Driven by population growth, ageing, and rising obesity, the global burden is projected to increase further, with knee OA cases expected to rise by approximately 75% by 2050 [2]. The knee is the most commonly affected joint, and no disease-modifying therapy is currently available for end-stage disease.

Total knee arthroplasty (TKA) remains the definitive treatment for end-stage knee OA and is among the most frequently performed orthopaedic procedures worldwide. Utilisation has increased substantially over recent decades, and national projections anticipate continued growth in both primary and revision procedures [3]. TKA reliably relieves pain and restores function, yet approximately one in five patients remains dissatisfied with the outcome [4], which has prompted renewed scrutiny of surgical technique and, in particular, of alignment strategy.

Systematic mechanical alignment, which targets a neutral hip–knee–ankle axis in every patient, has long been regarded as the reference standard and is supported by excellent long-term implant survivorship, although the degree to which coronal alignment itself determines outcome remains debated [5]. More anatomical strategies—kinematic, restricted kinematic, and functional alignment—instead aim to restore constitutional limb alignment and native joint line obliquity, and classification systems such as the Coronal Plane Alignment of the Knee (CPAK) have been developed to describe these constitutional phenotypes [6]. Comparative studies have shown that these strategies produce different joint line orientations at the knee, and some evidence suggests they may also influence alignment at the ankle [7,8].

This matters because the lower limb functions as a closed kinetic chain, in which the knee, ankle, subtalar joint, and hindfoot are mechanically coupled. Coronal malalignment at the knee alters the orientation of the load-bearing axis distally, and radiographic studies have shown that knee malalignment is directly reflected in ankle alignment in both varus and valgus gonarthrosis [9]. The subtalar joint is central to this coupling: three-dimensional in vivo analysis has demonstrated that it accommodates substantial inversion–eversion motion coupled with dorsi- and plantarflexion [10], allowing the hindfoot to act as the principal compensatory segment of the distal limb. Compensatory alignment of the subtalar joint has in turn been correlated with the progression of primary ankle osteoarthritis [11], and hindfoot alignment measured radiographically influences dynamic foot–floor pressure distribution [12].

In varus knee osteoarthritis, the hindfoot commonly adopts a compensatory valgus posture that partially offsets the varus tilt of the ankle joint line, and the magnitude and anatomical location of this compensation have been characterised in advanced disease [13]. Correction of knee alignment removes this demand abruptly, so that the ankle and subtalar joint must accommodate a new load vector within a short period. The consequences have been documented in several series: hindfoot alignment changes measurably after TKA [14,15], these changes are still present at one year [16], and compensatory adaptation after correction of varus deformity occurs not only at the ankle and subtalar joint but throughout the foot, with the magnitude of hindfoot change approximating half that of the change in limb mechanical alignment [17].

The capacity of the distal limb to absorb this change is not uniform. Hindfoot flexibility appears to determine the extent of compensation [18], and pre-existing severe knee deformity has been associated with persistent postoperative hindfoot pain and malalignment [19]. Concomitant ankle degeneration is common in this population, and ankle osteoarthritis has been shown to be associated with knee osteoarthritis [20]; its presence has been related to increased ankle pain and worse clinical outcomes after TKA [18], and it may limit the capacity of the ankle to adapt.

Clinical consequences have been described but remain inconsistent. Ankle pain has been reported in roughly one in four patients after TKA [21,22], whereas correction of varus malalignment by high tibial osteotomy has been reported to reduce ankle symptoms [23], and amelioration of ankle pain with improved function after TKA has been described in patients with combined ipsilateral knee and ankle osteoarthritis [24]. Aggravation of ankle varus incongruency appears to depend on the magnitude of correction, with a threshold of approximately 10° of varus deformity repeatedly implicated [25,26]. Beyond alignment, the ankle is also exposed to a second, largely unexamined influence: the axial forces transmitted distally during tibial preparation and component impaction.

Despite this, existing studies have relied almost exclusively on radiographic alignment parameters and patient-reported symptoms. The structural status of the ipsilateral ankle after TKA—the state of the articular cartilage, subchondral bone, and bone marrow—has not been characterised prospectively using magnetic resonance imaging (MRI), which is far more sensitive than radiography to early osteochondral and marrow change. Consequently, it is unknown whether the alignment changes and clinical improvements observed after TKA are accompanied by detectable structural changes at the ankle, and if so, whether such changes relate to the magnitude of alignment correction or to other perioperative factors. This is the knowledge gap the present study addresses.

The aim of this prospective study was to evaluate the clinical, radiographic, and MRI-detected structural changes in the ipsilateral ankle in patients undergoing primary TKA with a systematic neutral mechanical alignment strategy, and to test whether such changes are related to the magnitude of mechanical axis correction.

We hypothesised that (i) mechanical axis correction after TKA would be accompanied by a measurable change in talar tilt; (ii) the frequency of MRI-detected osteochondral findings and bone marrow oedema in the ipsilateral ankle would increase over the early postoperative period despite improvement in ankle-specific clinical scores; and (iii) the magnitude of mechanical axis correction would be associated with the extent of these radiographic and MRI changes.

## 2. Materials and Methods

### 2.1. Study Design and Participants

This single-centre, prospective observational study was conducted in the Department of Orthopaedics and Traumatology, Faculty of Medicine, Afyonkarahisar Health Sciences University, in collaboration with the Department of Radiology. The protocol was approved by the Non-Interventional Clinical Research Ethics Committee of Afyonkarahisar Health Sciences University Faculty of Medicine (decision no. 2023/9, dated 1 September 2023) and was conducted in accordance with the Declaration of Helsinki. Written informed consent was obtained from all participants prior to enrolment.

Consecutive patients undergoing primary TKA for end-stage knee osteoarthritis between 1 September 2023 and 1 September 2024 were screened. Inclusion criteria were age between 40 and 80 years and availability for outpatient follow-up. Exclusion criteria were a history of fracture, previous surgery, or significant deformity of the ipsilateral limb; known neurological deficit, peripheral neuropathy, or neuromuscular disease; and inability to undergo magnetic resonance imaging (MRI).

A total of 71 patients were enrolled, contributing 80 operated limbs. Nine patients underwent staged bilateral TKA during the study period, and both operated limbs of these patients were included in the imaging analyses.

**Unit of analysis.** Because bilateral cases were present, the unit of analysis was defined a priori for each outcome domain and is applied consistently throughout the manuscript. Radiological and MRI outcomes (mechanical axis, talar tilt, osteochondral findings, bone marrow oedema) were assessed separately for each operated limb and were therefore analysed at the limb level (*n* = 80); within-patient correlation arising from bilateral cases was accounted for using mixed-effects models and generalised estimating equations (Section 2.6). Clinical outcomes (FAOS) were administered once per patient rather than separately for each limb; to avoid duplication of identical patient-level scores across two limbs, clinical analyses were restricted to the first operated limb of each patient (*n* = 71).

### 2.2. Surgical Procedure

All procedures were performed by a single senior orthopaedic surgeon who completed specialty training in 2000 and thus had approximately 24 years of experience at the time of the study. A standardised technique was used in all cases. Surgery was performed under spinal anaesthesia with tourniquet control, through a medial parapatellar approach. Cemented posterior-stabilised (cruciate-sacrificing) implants (ADREMED WIND^®^ total knee prosthesis; Adremed, Ankara, Türkiye) were used in all cases, and the patella was resurfaced in every patient.

A systematic mechanical alignment strategy was applied, targeting a neutral hip–knee–ankle axis (0° ± 3°), with the distal femoral cut perpendicular to the femoral mechanical axis and the proximal tibial cut perpendicular to the tibial mechanical axis. A posterior tibial slope of 7° was used in all cases. Kinematic, restricted kinematic, and functional alignment techniques were not used. All patients followed an identical standardised rehabilitation protocol, with full weight-bearing permitted from the first postoperative day.

### 2.3. Clinical Assessment

Ankle-specific clinical status was evaluated using the Foot and Ankle Outcome Score (FAOS), an adaptation of the Knee Injury and Osteoarthritis Outcome Score comprising 42 items across five subscales: Pain (9 items), Other Symptoms (7 items), Activities of Daily Living (17 items), Sport and Recreation (4 items), and Foot- and Ankle-related Quality of Life (4 items). Each item is scored from 0 to 4; subscale raw scores are transformed to a 0–100 scale, with higher scores indicating fewer symptoms and better function [27]. The Turkish version of the FAOS, which has been formally validated for use in this population [28], was used throughout. Knee-specific function was assessed with the Hospital for Special Surgery (HSS) knee score.

FAOS and HSS were completed preoperatively and at the postoperative follow-up visit, on the same day as the corresponding radiographic and MRI assessments. Both instruments were administered once per patient at each time point rather than separately for each limb; the implications of this for the analysis are set out in Section 2.1 and in the Limitations.

### 2.4. Radiological and MRI Assessment

**Figure 1:**
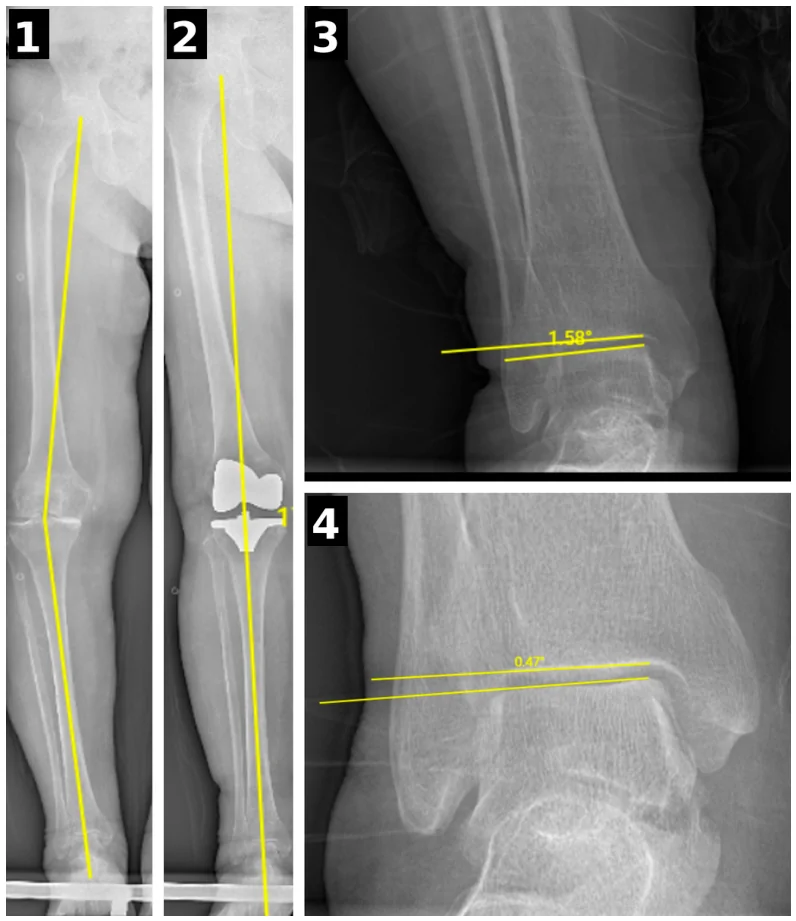
Preoperative and postoperative MA and TT measurements. (**1**): preoperative MA; (**2**): postoperative MA; (**3**): preoperative TT; (**4**): postoperative TT.

**Radiographic measurements.** The mechanical axis (MA) was measured on full-length standing weight-bearing anteroposterior teleroentgenograms (long-leg views) of the lower extremity as the angle between the femoral and tibial mechanical axes. Talar tilt (TT) was measured on weight-bearing anteroposterior ankle radiographs as the angle between the tibial plafond and the talar dome. Varus deviation was recorded as a positive value and valgus deviation as a negative value; the corresponding sign convention was applied to TT. Measurements were obtained preoperatively and at the postoperative follow-up visit. Representative preoperative and postoperative MA and TT measurements are shown in Figure 1.

**MRI protocol.** Ipsilateral ankle MRI was performed at three time points: preoperatively, in the early postoperative period (before discharge), and at the outpatient follow-up visit. The early postoperative examination was included to capture immediate perioperative changes and to distinguish them from findings evolving over the subsequent follow-up interval. All examinations were obtained on the same scanner throughout the study period using the institutional standard ankle protocol, which included T1- and T2-weighted sequences in the axial, coronal, and sagittal planes. Because the study used examinations acquired as part of routine clinical care, the scanner specifications and detailed acquisition parameters (repetition time, echo time, slice thickness, field of view) could not be retrieved retrospectively; this is acknowledged as a limitation.

**Image interpretation.** All MRI examinations were evaluated by a single radiologist who completed specialty training in 1998 and had approximately 26 years of experience, including extensive experience in musculoskeletal imaging. For each examination, the talar dome, distal tibia, and subtalar joint were assessed separately, and two categories of finding were recorded independently of one another: (i) chondral/osteochondral findings, including chondral thinning, osteochondral lesions, and subcortical cysts, recorded with their maximum dimension where measurable; and (ii) bone marrow oedema, defined as an ill-defined region of increased signal intensity within the subchondral or medullary bone on T2-weighted images, without a discrete cortical or chondral defect. Every examination was systematically assessed for both categories at all three anatomical sites. Because a single reader performed all interpretations, inter-observer reliability could not be calculated, and the reader was not blinded to the time point of acquisition; both issues are acknowledged as limitations.

**Reporting convention.** Reports were generated as part of routine clinical practice, and a finding documented at an earlier examination was not necessarily restated in subsequent reports. Consequently, the absence of a finding at a later time point cannot reliably be interpreted as radiological resolution. To address this, findings are presented both as observed at each time point and as cumulative incidence of newly reported findings, and a carry-forward sensitivity analysis was performed (Section 2.6).

### 2.5. Outcomes

The primary outcome was the change in MRI-detected chondral/osteochondral findings and bone marrow oedema in the ipsilateral ankle across the three time points. Secondary outcomes were the change in MA and TT, the change in FAOS and HSS scores, and the association between the magnitude of mechanical axis correction (ΔMA) and changes in TT, FAOS, and the development of newly reported MRI findings.

### 2.6. Statistical Analysis

Continuous variables are presented as mean ± standard deviation (SD) and median (interquartile range, IQR) with minimum–maximum values; categorical variables are presented as counts and percentages. Follow-up duration is reported as median (IQR) and range.

No a priori sample size calculation was performed. All consecutive patients meeting the eligibility criteria within the predefined 12-month recruitment window were included, and the resulting precision is reflected in the confidence intervals reported throughout. Analyses were conducted on the complete dataset; no imputation was performed for missing values other than the carry-forward sensitivity analysis described below, which was applied only to the binary MRI outcomes. All measurements were extracted directly from the source clinical records and verified against them before analysis.

Because outcomes were measured repeatedly within the same limbs and because nine patients contributed two limbs, statistical methods appropriate for clustered and repeated-measures data were used throughout; tests assuming independent observations were not applied to longitudinal comparisons.

Continuous limb-level outcomes (MA, TT) were analysed using linear mixed-effects models with time point as a fixed effect and patient as a random intercept. Mean differences are reported with 95% confidence intervals (CI), together with Cohen’s d for paired data.

Clinical outcomes (FAOS, HSS), analysed at the patient level (*n* = 71), were compared between time points using the Wilcoxon signed-rank test, with mean changes, 95% confidence intervals, and standardised effect sizes reported.

Binary MRI outcomes were analysed using logistic generalised estimating equations (GEE) with patient as the clustering variable and an exchangeable working correlation structure, yielding odds ratios (OR) with 95% confidence intervals relative to the preoperative examination. Paired within-limb transitions were additionally examined using the exact McNemar test. Because previously documented findings were not systematically restated in later reports, the cumulative incidence of newly reported findings among limbs free of that finding preoperatively is presented as the primary descriptive measure, and a carry-forward sensitivity analysis (in which any finding once reported was assumed to persist) was performed. As the carry-forward assumption permits prevalence only to increase, its significance tests are reported for completeness but are not interpreted as formal hypothesis tests.

Associations with the magnitude of alignment correction were assessed by correlating ΔMA with ΔTT, ΔFAOS, and ΔHSS (Pearson and Spearman coefficients), and by modelling the development of newly reported MRI findings as a function of ΔMA using logistic GEE. The incidence of MRI findings was additionally stratified by tertiles of ΔMA. Follow-up duration was examined as a potential confounder.

A two-sided *p* value < 0.05 was considered statistically significant. Analyses were performed using Python 3.12 (Python Software Foundation, Wilmington, DE, USA) with the statsmodels, SciPy, and pandas libraries.

## 3. Results

### 3.1. Participants

**Figure 2:**
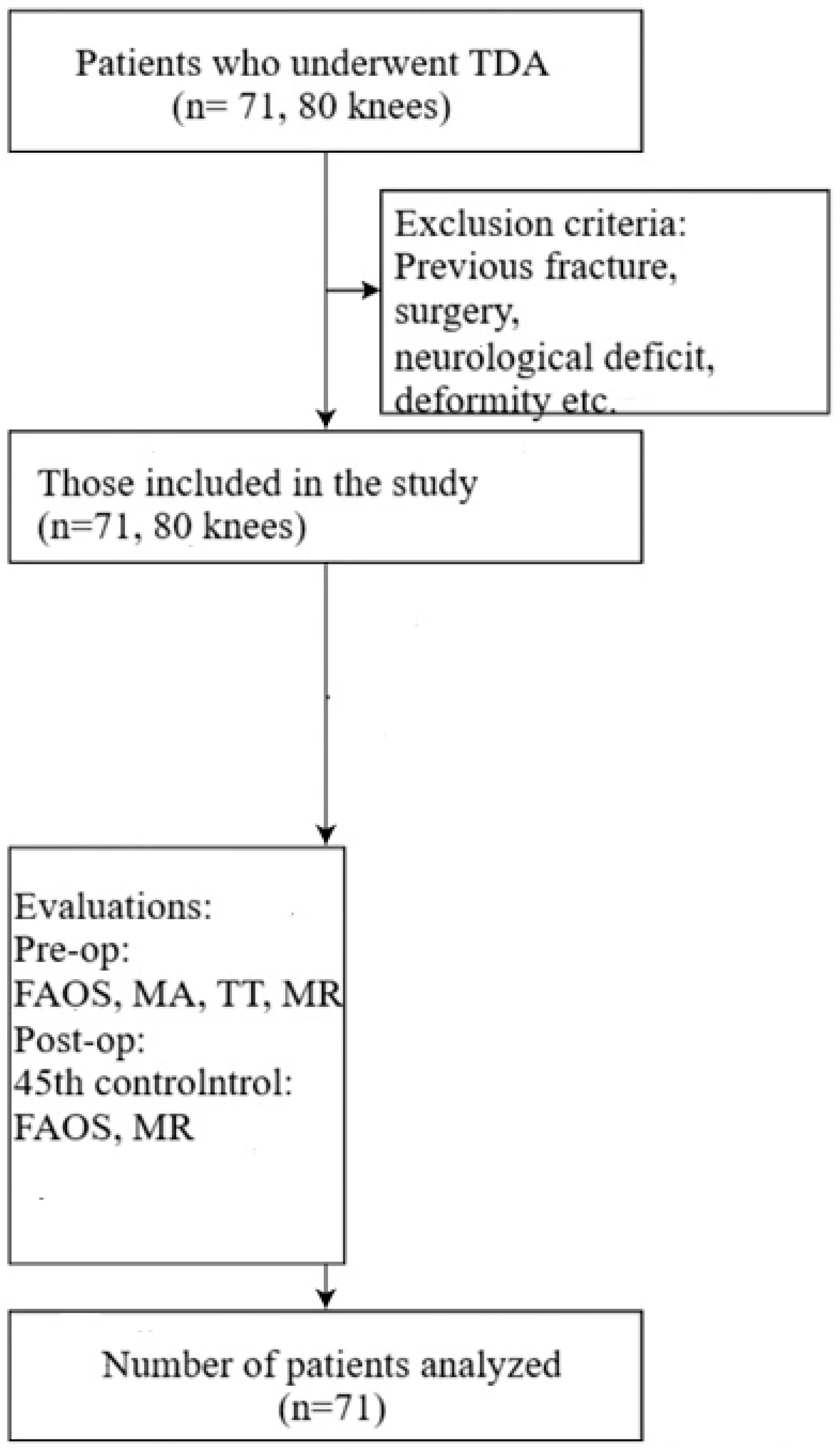
Flow chart of the study.

Seventy-one patients contributing 80 operated limbs were included; nine patients underwent staged bilateral TKA. The mean age was 63.9 ± 7.2 years (range 50–80), and 59 patients (83.1%) were female. Surgery was performed on the left knee in 42 limbs (52.5%) and on the right knee in 38 limbs (47.5%) (Table 1). The flow of participants through the study is shown in Figure 2.

The follow-up assessment was scheduled at approximately 45 days. The observed interval between surgery and follow-up was median 37 days (IQR 34–44; range 19–198; mean 43.8 ± 30.7). This variability reflected the constraints of routine outpatient scheduling: eleven limbs (13.8%) were reassessed before day 30 and five (6.3%) after day 60, the latter mainly in patients whose appointments were deferred for logistical reasons. Follow-up duration was not associated with the presence of MRI findings at follow-up (r = −0.152, *p* = 0.179) and was examined as a covariate in the analyses reported below.

### 3.2. Radiographic Alignment

**Figure 3:**
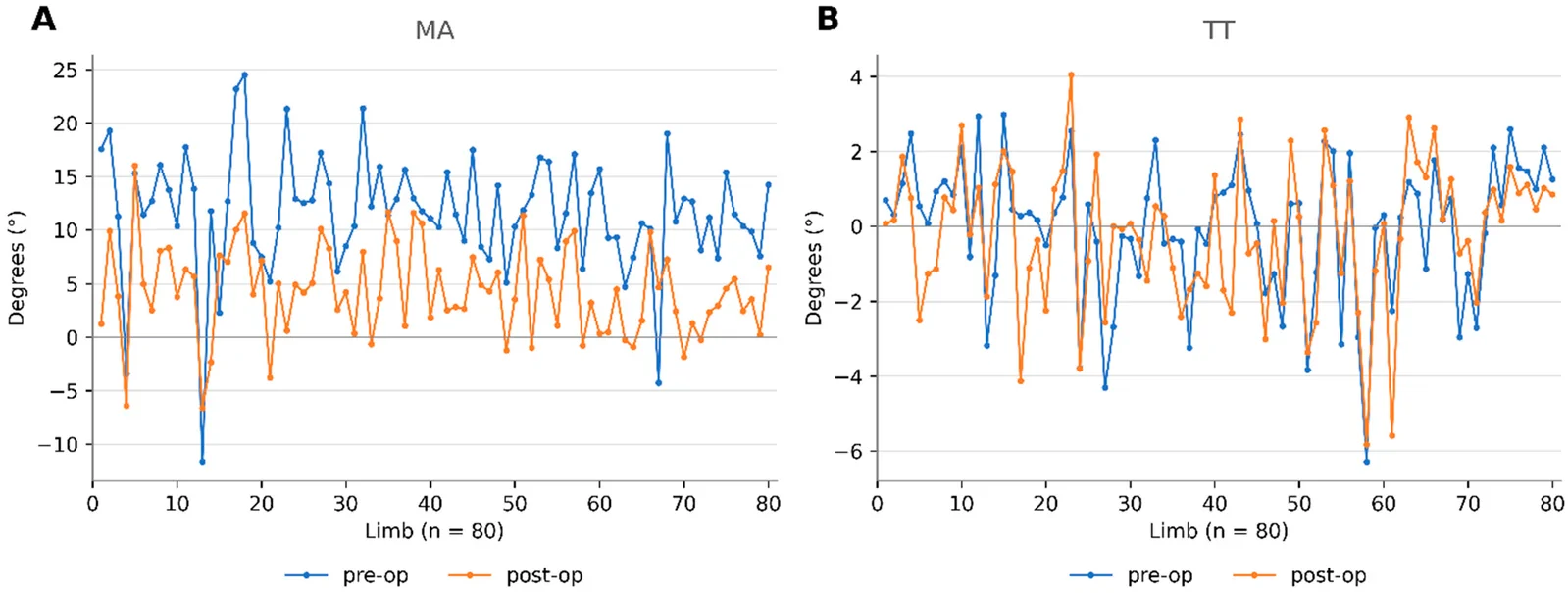
(**A**) Preoperative and postoperative MA values; (**B**) preoperative and postoperative TT values.

Preoperatively, 77 limbs (96.3%) were in varus and 3 (3.8%) in valgus alignment. The mechanical axis improved markedly after surgery, whereas talar tilt remained essentially unchanged (Table 2, Figure 3).

A mean correction of 7.3° at the knee was therefore achieved without a corresponding change in tibiotalar joint line orientation.

### 3.3. Clinical Outcomes

All FAOS subscales improved significantly between the preoperative and follow-up assessments (Table 3).

No patient reported new ankle symptoms requiring treatment during the follow-up period.

### 3.4. MRI Findings

**Figure 4:**
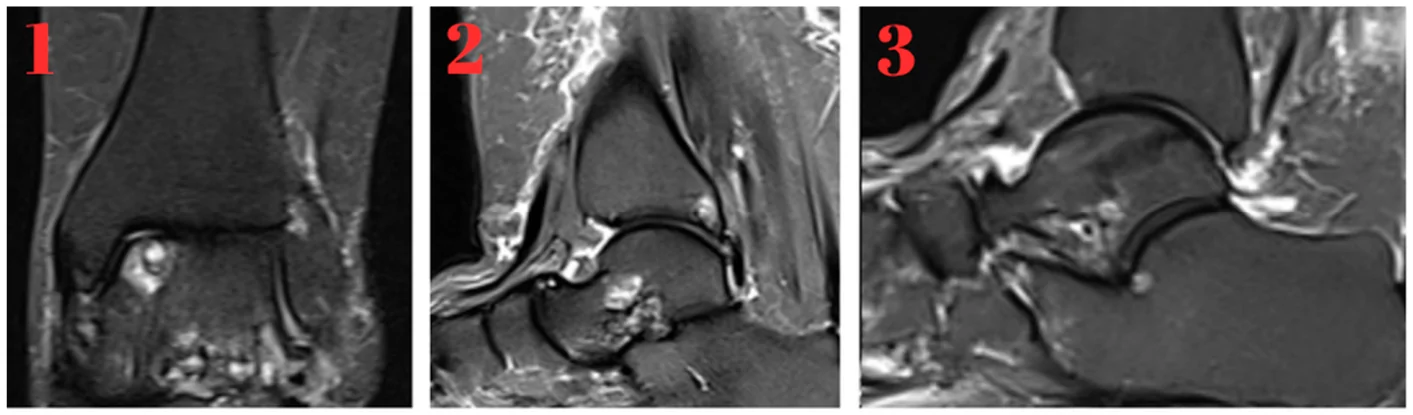
Representative MRI findings in the ipsilateral ankle. (**1**): osteochondral finding of the talus (coronal T2-weighted image); (**2**): osteochondral finding of the distal tibia (sagittal T2-weighted image); (**3**): subcortical cyst at the subtalar joint (sagittal T2-weighted image).

Chondral or osteochondral findings were already present in 33 limbs (41.2%) preoperatively. The prevalence of findings at each time point and the cumulative incidence of newly reported findings are shown in Table 4. Representative MRI findings are shown in Figure 4.

Newly reported bone marrow oedema was concentrated in the distal tibia, occurring in 16 of 79 limbs at risk (20.3%), approximately twice the frequency observed at the talus (8/79, 10.1%) and more than twice that at the subtalar joint (6/70, 8.6%). Prevalence of distal tibial oedema rose from 1.2% preoperatively to 8.8% in the early postoperative period and 13.8% at follow-up (GEE OR 8.18, 95% CI 1.68–39.89, *p* = 0.009 and OR 13.73, 95% CI 1.54–122.71, *p* = 0.019, respectively; exact McNemar *p* = 0.006 for the preoperative-to-follow-up transition). Because preoperative prevalence was very low, the confidence intervals around these odds ratios are wide and the magnitude of the effect should be interpreted with caution.

Chondral/osteochondral findings in the distal tibia showed a parallel but weaker increase, from 10.0% to 18.8% at follow-up (OR 2.05, 95% CI 1.01–4.19, *p* = 0.048). Subtalar bone marrow oedema did not increase at any time point, and no significant change over time was observed at the talus for either category of finding. Considered across all sites together, neither category reached statistical significance: chondral/osteochondral findings increased from 41.2% preoperatively to 51.2% at follow-up (OR 1.50, 95% CI 0.94–2.38, *p* = 0.088) and bone marrow oedema from 15.0% to 23.8% (OR 1.77, 95% CI 0.88–3.58, *p* = 0.111).

In the carry-forward sensitivity analysis (Table 5), in which any finding once documented was assumed to persist, the prevalence of chondral/osteochondral findings at follow-up rose to 45 limbs at the talus, 20 at the distal tibia, 17 at the subtalar joint, and 52 at any site; corresponding figures for bone marrow oedema were 9, 17, 16, and 33. As this assumption permits prevalence only to increase, the associated significance tests are not interpreted as formal hypothesis tests; the analysis is presented to define the upper bound of the possible change.

### 3.5. Association Between Alignment Correction and Ankle Outcomes

The magnitude of mechanical axis correction (ΔMA) was not associated with any ankle outcome (Table 6). Correlations were negligible for ΔTT (r = −0.040, *p* = 0.724; Spearman ρ = −0.054, *p* = 0.634), ΔFAOS total (r = 0.023, *p* = 0.851), and ΔHSS (r = 0.032, *p* = 0.792). Among limbs without a preoperative finding (*n* = 47), the development of a newly reported MRI finding was unrelated to ΔMA (logistic GEE: OR 1.021 per degree, 95% CI 0.923–1.131, *p* = 0.682), as was the development of new bone marrow oedema (OR 0.966 per degree, 95% CI 0.846–1.102, *p* = 0.608).

Stratification by tertiles of ΔMA showed no dose–response relationship (Table 7). The prevalence of MRI findings at follow-up was 59% in the tertile with the largest correction (mean ΔMA −12.3°), 31% in the middle tertile (−7.6°), and 63% in the tertile with the smallest correction (−2.2°).

### 3.6. Clinical–Imaging Relationship

Patients in whom a new chondral or osteochondral finding was reported at follow-up showed no meaningful improvement in FAOS total score (+0.3 points, *n* = 14), whereas those without a new finding improved substantially (+21.9 points, *n* = 57; *p* = 0.044). In contrast, newly reported bone marrow oedema was not associated with FAOS change (+23.1 vs. +15.8 points, *p* = 0.397). Given the small subgroup sizes, the number of comparisons performed, and the borderline *p* value, these observations should be regarded as hypothesis-generating.

## 4. Discussion

The principal findings of this prospective study are threefold. First, TKA performed with a systematic neutral mechanical alignment strategy produced a mean correction of 7.3° in the mechanical axis without a corresponding change in tibiotalar joint line orientation, while ankle-specific and knee-specific clinical scores improved significantly. Second, MRI abnormalities in the ipsilateral ankle were newly reported over the early follow-up period predominantly in the distal tibia, and bone marrow oedema showed the clearest anatomical concentration. Third, and contrary to the prevailing biomechanical hypothesis, neither the development of newly reported MRI findings nor the change in talar tilt was associated with the magnitude of mechanical axis correction.

### 4.1. Alignment and the Ankle

Compensatory hindfoot and ankle alignment changes after correction of knee varus deformity are well described. Mullaji et al. first documented that hindfoot valgus decreases after TKA for varus deformity while residual deformity persists [29]. Chang et al. reported that the ankle joint line returned from 9.4° to 3.4° varus after TKA with a 2.2° reduction in hindfoot valgus compensation, and that hindfoot flexibility was lower in patients with concomitant ankle osteoarthritis [18]. Hindfoot alignment is generally reported to change by approximately half the magnitude of the change in lower limb mechanical alignment [17].

In the present cohort, mechanical alignment improved substantially (11.62° to 4.29° varus), whereas talar tilt was essentially unchanged (−0.02° to −0.22°, *p* = 0.256). Notably, aggravation of ankle varus incongruency after TKA has been consistently linked to a correction threshold of approximately 10° of varus deformity [25,26]. The mean correction of 7.3° achieved in our cohort falls below this threshold, which may explain the absence of a measurable change in talar tilt. This interpretation is consistent with earlier reports in which tibiotalar tilt was not significantly altered after TKA despite improvement in tibial plafond and talar inclination [30,31]. An alternative explanation is that compensation after TKA occurs predominantly at the subtalar joint and hindfoot rather than at the tibiotalar joint line, which talar tilt captures. Radiographic assessment of hindfoot alignment and flexibility was not performed in this study, which limits mechanistic interpretation.

### 4.2. Clinical Outcomes

Ankle symptoms after TKA have been reported in approximately one quarter of patients and have been associated with preoperative talar inclination and lateralised gait rather than with joint laxity or range of motion [21]. Ankle pain has also been described in 22.8% of patients in relation to component positioning parameters [22], and in 11.4% of patients over a mean 13.2-month follow-up after bilateral TKA for severe varus deformity [4]. Conversely, correction of varus malalignment by high tibial osteotomy has been shown to reduce ankle pain and improve function [23].

Our findings are consistent with the latter pattern: all FAOS subscales improved significantly, with the largest gains in sport and recreation and quality of life, and no patient developed new ankle symptoms requiring treatment. Together with the marked improvement in knee-specific function, this indicates that correction of varus malalignment was clinically well tolerated at the ankle in the early postoperative period.

### 4.3. MRI Findings and Their Interpretation

The most informative aspect of the imaging data is its anatomical specificity. Newly reported bone marrow oedema occurred in the distal tibia at roughly twice the frequency seen at the talus and the subtalar joint, and prevalence of distal tibial oedema rose from 1.2% preoperatively to 13.8% at follow-up. Chondral and osteochondral findings in the distal tibia showed a parallel, weaker increase. In contrast, subtalar oedema did not increase at any time point.

This distribution is difficult to reconcile with a mechanism based on redistribution of load vectors, which would be expected to affect the subtalar joint and hindfoot preferentially [18,32]. It is more consistent with a perioperative, tibia-localised process. Bone marrow oedema of this pattern and timing is compatible with the physiological response to impaction and instrumentation during tibial preparation. Prissel et al. found no relationship between bone oedema and pain after total ankle replacement, with only subtalar oedema relating to reoperation and survivorship [33], while preoperative subchondral bone marrow oedema at the knee has been associated with greater postoperative pain after arthroplasty [34].

Importantly, chondral and osteochondral findings were already present in 41.2% of limbs preoperatively, consistent with the recognised association between ankle and knee osteoarthritis in this population [20]. We therefore do not interpret our findings as demonstrating the de novo formation of osteochondral lesions within this short interval, which would be biologically implausible; rather, they represent findings newly identified at follow-up in a population with a high baseline burden of ankle degeneration. Bone marrow oedema and chondral/osteochondral change were recorded as separate categories throughout, so the reported osteochondral findings do not incorporate oedema-like signal, although the absence of fat-suppressed sequences limits the precision of this distinction.

### 4.4. Alignment Magnitude and Mechanistic Implications

The magnitude of correction has repeatedly been implicated as the determinant of ankle deterioration after TKA, with aggravation of ankle varus incongruency linked to corrections of approximately 10° or more [25,26]. A comparable relationship has been reported after correction of severe valgus deformity, where larger corrections were associated with increased postoperative ankle symptoms and reduced subtalar mobility correlated strongly with symptom worsening [32]; that cohort, however, differs from ours, in which 96.3% of limbs were in varus preoperatively. Our mean correction of 7.3° falls below the reported varus threshold, which may explain the absence of clinical ankle deterioration in this cohort.

Critically, we tested the alignment hypothesis directly rather than inferring it. The magnitude of correction was unrelated to the change in talar tilt (r = −0.040), to the change in FAOS (r = 0.023), and to the development of newly reported MRI findings (OR 1.021 per degree, *p* = 0.682) or new bone marrow oedema (OR 0.966 per degree, *p* = 0.608). Stratification by tertiles of correction showed no dose–response gradient. Demonstrating that alignment and imaging findings both change over time does not establish a relationship between them, and when tested explicitly, no such relationship was evident in this cohort.

These observations bear on the ongoing debate between systematic mechanical alignment and more anatomical strategies such as kinematic, restricted kinematic, and functional alignment, and on classification systems describing constitutional coronal phenotypes [6]. Kinematically aligned TKA has been reported to restore ankle joint line orientation closer to that of the native ankle than mechanical alignment [7]. However, in a randomised controlled trial comparing functional and mechanical alignment within the same patient, postoperative ankle joint line orientation did not differ significantly between the two strategies except in one specific coronal phenotype [8]. Our data neither support nor refute these observations: all patients in this cohort were treated with a single systematic neutral mechanical alignment target, so no comparison between alignment philosophies was possible. The absence of any relationship between correction magnitude and ankle findings nonetheless suggests that, within the range of correction studied, alignment strategy may be less relevant to the early ankle response than perioperative factors. Prospective comparison of alignment philosophies with ankle imaging endpoints would be required to address this question.

### 4.5. Clinical–Imaging Relationship

An apparent dissociation between clinical and imaging outcomes was observed: FAOS scores improved substantially while MRI abnormalities were newly reported in a substantial proportion of limbs. Exploratory subgroup analysis suggested that this dissociation was not uniform. Patients in whom a new chondral or osteochondral finding appeared showed essentially no improvement in FAOS total score (+0.3 points, *n* = 14), whereas those without a new finding improved markedly (+21.9 points, *n* = 57; *p* = 0.044). Newly reported bone marrow oedema, by contrast, showed no relationship with clinical change (*p* = 0.397), suggesting that oedema in this setting is clinically silent in the short term.

Because of small subgroup sizes, multiple comparisons, and a borderline *p* value, these observations are hypothesis-generating rather than confirmatory. They nonetheless suggest that the clinical–imaging dissociation reported after TKA may depend on the type of imaging finding, and that this distinction merits prospective evaluation with longer follow-up.

### 4.6. Limitations

This study has several important limitations. There was no control group of patients not undergoing TKA, so the observed imaging changes cannot be distinguished from the natural progression of pre-existing ankle degeneration. The follow-up period was short and highly variable (median 37 days, range 19–198), which precludes assessment of the clinical significance and long-term evolution of the imaging findings; although follow-up duration was not associated with imaging findings, residual confounding cannot be excluded. The sample size was limited and the study was conducted at a single centre with a single surgeon, which enhances technical homogeneity but restricts generalisability. Nine patients contributed both limbs; although within-patient correlation was accounted for statistically, these observations are not fully independent. FAOS and HSS were administered once per patient rather than separately for each limb, so clinical analyses were restricted to one limb per patient.

MRI reports were generated as part of routine clinical care, and previously documented findings were not systematically restated at subsequent examinations. The absence of a finding at a later time point therefore cannot be interpreted as radiological resolution, and prevalence estimates at individual time points should be read with this in mind; for this reason the cumulative incidence of newly reported findings, together with a carry-forward sensitivity analysis, is presented alongside observed prevalence.

Bone marrow oedema was identified on T2-weighted images; fat-suppressed fluid-sensitive sequences, which are more sensitive and more specific for marrow oedema, were not part of the institutional ankle protocol. Subtle oedema may therefore have been under-detected, and the reported frequencies should be regarded as conservative estimates. All MRI examinations were interpreted by a single experienced radiologist who was not blinded to the time point of acquisition; consequently, inter-observer reliability could not be calculated and observer bias cannot be excluded. Findings were not graded using a validated classification system such as the modified Whole-Organ Magnetic Resonance Imaging Score (WORMS), Hepple, or International Cartilage Repair Society (ICRS) systems [35,36], and scanner specifications and detailed sequence parameters could not be retrieved retrospectively. Radiographic assessment of hindfoot alignment, hindfoot flexibility, and subtalar mobility was not performed, limiting evaluation of compensatory mechanisms. Intraoperative forces were not measured, so no mechanistic claim regarding impaction can be substantiated. Finally, the observational design does not permit causal inferences regarding the relationships between TKA, alignment correction, and ankle imaging findings.

### 4.7. Strengths

This study has several methodological strengths. It is, to our knowledge, the first prospective series to characterise the ipsilateral ankle after TKA with serial MRI at three defined time points, capturing both chondral/osteochondral status and bone marrow signal rather than radiographic alignment alone. All procedures were performed by a single surgeon using a single implant, a single approach, and one uniform alignment target, which removes technique and implant heterogeneity as sources of variation and makes the observed anatomical concentration of findings easier to interpret. All imaging was interpreted by one experienced musculoskeletal radiologist using a single institutional protocol, so that inter-reader variation does not contribute to the observed changes, and chondral/osteochondral findings and bone marrow oedema were recorded as separate categories from the outset rather than being disentangled retrospectively. Finally, the central hypothesis was tested directly rather than inferred, and the analysis accounts explicitly for the clustered structure of the data.

### 4.8. Clinical Implications and Future Directions

Three practical implications follow from these findings. First, within a correction range typical of routine varus TKA, patients can be reassured that ankle-specific symptoms improve rather than deteriorate in the early postoperative period, and that improvement occurs across all FAOS domains. Second, MRI abnormalities detected in the ipsilateral ankle shortly after TKA should be interpreted with caution: a substantial proportion of patients in this population carry pre-existing ankle degeneration, and a newly documented finding at six weeks is more likely to represent detection than formation. Third, the anatomical concentration of change in the distal tibia, particularly of bone marrow oedema, suggests that if surveillance imaging is undertaken it should include the distal tibia and not be confined to the talar dome, which has been the traditional focus of osteochondral assessment.

The interdependence of the two joints is bidirectional and is therefore relevant beyond the setting of knee arthroplasty: concomitant ipsilateral knee pain has been shown to impair functional outcomes after total ankle arthroplasty [37], and the structural condition of the distal tibia and talus influences the management and reconstruction options available should ankle surgery later become necessary [38]. This reinforces the argument for assessing the ankle as part of the routine evaluation of the arthritic knee rather than as an afterthought.

Several priorities for future work follow directly from the limitations of this study. Longer follow-up is required to establish whether the imaging changes observed here persist, resolve, or progress, and whether the absence of early clinical consequence is maintained; a follow-up interval of one to two years would permit this while remaining within the window in which compensatory hindfoot adaptation is known to occur. Prospective studies should incorporate fat-suppressed fluid-sensitive sequences with standardised acquisition parameters, apply a validated grading system such as the modified WORMS, Hepple, or ICRS classifications [35,36], and use at least two blinded readers so that inter-observer reliability can be quantified. Baseline assessment of hindfoot alignment, hindfoot flexibility, and subtalar mobility would allow the stratification that our data could not support, and would test directly whether patients with a rigid hindfoot are those in whom alignment correction is transmitted to the ankle. A control group of patients with knee osteoarthritis managed non-operatively would separate perioperative change from the natural history of pre-existing ankle degeneration. Finally, because our cohort was treated exclusively with a systematic neutral mechanical alignment target, comparative studies of mechanical, restricted kinematic, and functional alignment with ankle imaging endpoints are needed to determine whether alignment philosophy influences the distal articular response.

The finding that requires the most careful replication is the apparent difference between finding types in their clinical correlates: newly reported chondral or osteochondral findings were accompanied by an absence of clinical improvement, whereas bone marrow oedema was not. If confirmed in a larger series with validated grading, this distinction would have direct implications for how incidental ankle MRI findings after TKA are communicated to patients.

## 5. Conclusions

Following TKA performed with a neutral mechanical alignment strategy, mechanical axis correction and clinical improvement were achieved without deterioration in ankle symptoms in the early postoperative period. MRI-detected osteochondral and bone marrow changes in the ipsilateral ankle were observed alongside these improvements; these changes were concentrated in the distal tibia and were not associated with the magnitude of alignment correction. Given the high preoperative prevalence of ankle degeneration in this population and the short follow-up interval, the mechanisms underlying these findings and their long-term clinical significance remain uncertain and warrant prospective study with longer follow-up and validated lesion grading.

## Figures and Tables

**Table 1:** Demographic and follow-up characteristics of the study population.

Variable	Mean ± SD	Median (IQR)	Min–Max
Age (years), patient level (*n* = 71)	63.86 ± 7.21	64 (58–69)	50–80
Interval to follow-up (days), limb level (*n* = 80)	43.8 ± 30.7	37 (34–44)	19–198
**Variable**	**Category**	***n***	**%**
Sex (patients, *n* = 71)	Female	59	83.1
	Male	12	16.9
Operated side (limbs, *n* = 80)	Left	42	52.5
	Right	38	47.5
Bilateral procedures	Patients	9	12.7

SD, standard deviation; IQR, interquartile range. Age and sex are reported at patient level; side and follow-up interval at limb level.

**Table 2:** Mechanical axis and talar tilt before and after TKA (*n* = 80 limbs).

Parameter	Preoperative	Follow-Up	Mean Change (95% CI)	*p*	d
MA (°)	11.62 ± 5.57	4.29 ± 4.27	−7.34 (−8.49 to −6.18)	<0.001	−1.48
TT (°)	−0.02 ± 1.87	−0.22 ± 1.91	−0.20 (−0.56 to +0.15)	0.256	−0.14

TKA, total knee arthroplasty; MA, mechanical axis; TT, talar tilt; CI, confidence interval; d, Cohen’s d for paired data. Linear mixed-effects models with patient as a random intercept. Positive values denote varus, negative values valgus.

**Table 3:** FAOS and HSS scores before and after TKA (*n* = 71 patients).

Score	Preoperative	Follow-Up	Mean Change (95% CI)	*p*	d
FAOS Symptoms	74.9 ± 24.3	90.9 ± 18.0	+16.0 (+9.2 to +22.8)	<0.001	0.55
FAOS Pain	70.2 ± 24.8	82.9 ± 27.8	+12.7 (+4.6 to +20.9)	0.004	0.36
FAOS ADL	66.5 ± 28.6	82.1 ± 28.7	+15.6 (+6.6 to +24.6)	0.002	0.40
FAOS Sport/Rec.	59.9 ± 36.4	82.3 ± 29.5	+22.5 (+12.1 to +32.8)	<0.001	0.50
FAOS QOL	62.1 ± 33.0	83.7 ± 28.9	+21.6 (+11.9 to +31.2)	<0.001	0.52
FAOS total	66.7 ± 27.6	84.4 ± 25.1	+17.7 (+9.5 to +25.8)	<0.001	0.50
HSS knee score	59.2 ± 9.8	76.6 ± 9.3	+17.5 (+14.9 to +20.0)	<0.001	1.57

TKA, total knee arthroplasty; FAOS, Foot and Ankle Outcome Score; ADL, activities of daily living; QOL, quality of life; HSS, Hospital for Special Surgery. Wilcoxon signed-rank test. Higher scores indicate better outcomes.

**Table 4:** MRI findings in the ipsilateral ankle across the three time points (*n* = 80 limbs).

Finding	Site	Preop *n* (%)	Early Postop *n* (%)	Follow-Up *n* (%)	Newly Reported *n*/at Risk (%)
Chondral/osteochondral	Talus	27 (33.8)	24 (30.0)	31 (38.8)	18/53 (34.0)
	Distal tibia	8 (10.0)	9 (11.2)	15 (18.8)	12/72 (16.7)
	Subtalar joint	8 (10.0)	10 (12.5)	11 (13.8)	9/72 (12.5)
	Any site	33 (41.2)	34 (42.5)	41 (51.2)	19/47 (40.4)
Bone marrow oedema	Talus	1 (1.2)	3 (3.8)	6 (7.5)	8/79 (10.1)
	Distal tibia	1 (1.2)	7 (8.8)	11 (13.8)	16/79 (20.3)
	Subtalar joint	10 (12.5)	7 (8.8)	7 (8.8)	6/70 (8.6)
	Any site	12 (15.0)	17 (21.2)	19 (23.8)	21/68 (30.9)

MRI, magnetic resonance imaging. “Newly reported” denotes a finding documented at the early postoperative or follow-up examination in a limb in which that finding was not documented preoperatively. Because previously documented findings were not systematically restated in later reports, prevalence at a given time point may underestimate the true burden; the newly reported column is therefore the more reliable measure of change.

**Table 5:** Carry-forward sensitivity analysis of MRI findings at follow-up (*n* = 80 limbs).

Finding	Site	Observed at Follow-Up *n* (%)	Carry-Forward *n* (%)	Difference
Chondral/osteochondral	Talus	31 (38.8)	45 (56.2)	14
	Distal tibia	15 (18.8)	20 (25.0)	5
	Subtalar joint	11 (13.8)	17 (21.2)	6
	Any site	41 (51.2)	52 (65.0)	11
Bone marrow oedema	Talus	6 (7.5)	9 (11.2)	3
	Distal tibia	11 (13.8)	17 (21.2)	6
	Subtalar joint	7 (8.8)	16 (20.0)	9
	Any site	19 (23.8)	33 (41.2)	14

MRI, magnetic resonance imaging. In the carry-forward analysis, any finding documented at any earlier examination was assumed to persist at follow-up. Because this assumption permits prevalence only to increase, the resulting values define the upper bound of the possible change and the associated significance tests are not interpreted as formal hypothesis tests. The observed and carry-forward values bracket the true prevalence at follow-up.

**Table 6:** Association between the magnitude of mechanical axis correction (ΔMA) and ankle outcomes.

Outcome	*n*	Test	Estimate (95% CI)	*p*
Δ Talar tilt	80	Pearson r	−0.040	0.724
Δ Talar tilt	80	Spearman ρ	−0.054	0.634
Δ FAOS total	71	Pearson r	0.023	0.851
Δ HSS knee score	71	Pearson r	0.032	0.792
Newly reported MRI finding	47	Logistic GEE, OR per degree	1.021 (0.923–1.131)	0.682
Newly reported bone marrow oedema	68	Logistic GEE, OR per degree	0.966 (0.846–1.102)	0.608

ΔMA, change in mechanical axis; FAOS, Foot and Ankle Outcome Score; HSS, Hospital for Special Surgery; GEE, generalised estimating equations; OR, odds ratio; CI, confidence interval. Binary outcomes were modelled among limbs in which the corresponding finding was absent preoperatively.

**Table 7:** MRI findings stratified by tertile of mechanical axis correction.

Tertile of ΔMA	*n* (Limbs)	Mean ΔMA (°)	Any Finding at Follow-Up *n* (%)	Newly Reported Finding *n*
Largest correction	27	−12.3	16 (59.3)	6
Intermediate	26	−7.6	8 (30.8)	1
Smallest correction	27	−2.2	17 (63.0)	7

MRI, magnetic resonance imaging; ΔMA, change in mechanical axis. No monotonic dose–response relationship was observed across tertiles of correction. Negative values denote correction of varus towards neutral.

## Data Availability

The datasets generated and/or analyzed during the current study are not publicly available due to patient confidentiality and institutional restrictions but are available from the corresponding author on reasonable request.

## Abbreviations

ADL, activities of daily living; CI, confidence interval; CPAK, Coronal Plane Alignment of the Knee; FAOS, Foot and Ankle Outcome Score; GEE, generalised estimating equations; HSS, Hospital for Special Surgery; ICRS, International Cartilage Repair Society; IQR, interquartile range; MA, mechanical axis; MRI, magnetic resonance imaging; OA, osteoarthritis; OR, odds ratio; QOL, quality of life; SD, standard deviation; TKA, total knee arthroplasty; TT, talar tilt; WORMS, Whole-Organ Magnetic Resonance Imaging Score.
